# The multiple actions of grape and its polyphenols on female reproductive processes with an emphasis on cell signalling

**DOI:** 10.3389/fendo.2023.1245512

**Published:** 2024-01-04

**Authors:** Ladislav Kohut, Simona Baldovska, Michal Mihal, Lubomir Belej, Alexander V. Sirotkin, Shubhadeep Roychoudhury, Adriana Kolesarova

**Affiliations:** ^1^ Institute of Applied Biology, Slovak University of Agriculture in Nitra, Nitra, Slovakia; ^2^ AgroBioTech Research Center, Slovak University of Agriculture in Nitra, Nitra, Slovakia; ^3^ Institute of Food Sciences, Slovak University of Agriculture in Nitra, Nitra, Slovakia; ^4^ Department of Zoology and Anthropology, Constantine the Philosopher University, Nitra, Slovakia; ^5^ Department of Life Science and Bioinformatics, Assam University, Silchar, India

**Keywords:** grapeseed extract, phenolic compounds, proanthocyanidin, resveratrol, delphinidin, female reproduction, steroid hormones, proliferation

## Abstract

Grapes are an economically important fruit crop, and their polyphenols (mainly phenolic acids, flavanols, flavonols, anthocyanins, proanthocyanidins, and stilbenes) can exert a wide range of health benefits as an interesting and valuable dietary supplement for natural complementary therapy. However, their potential physiological and therapeutic actions on reproductive processes have not been sufficiently elucidated. This evidence-based study presents current knowledge of grape extracts and polyphenols, as well as their properties and therapeutical actions in relation to female reproduction in a nutshell. Grape extract, and its polyphenols such as resveratrol, proanthocyanidin B2 or delphinidin may influence female reproductive physiology and pathology, as well as regulate multiple signaling pathways related to reproductive hormones, steroid hormones receptors, intracellular regulators of oxidative stress and subsequent inflammation, apoptosis, and proliferation. Their role in the management of ovarian cancer, age-related reproductive insufficiency, ovarian ischemia, PCOS, or menopausal syndrome has been indicated. In particular, the potential involvement of grapeseed extracts and/or proanthocyanidin B2 and delphinidin on ovarian steroidogenesis, oocyte maturation, and developmental capacity has been implicated, albeit at different regulatory levels. Grape polyphenols exert a wide range of health benefits posing grape extract as an interesting and valuable dietary supplement for natural complementary therapy. This evidence-based study focuses on the actions of grapeseed extract and grape polyphenols on female reproductive processes at various regulatory levels and multiple signalling pathways by regulating reproductive hormones (GnRH, gonadotropins, prolactin, steroid hormones, IGFBP), steroid receptors, markers of proliferation and apoptosis. However, lack of knowledge of standardized dosages so far limits their clinical application despite the wide range of their biological and therapeutic potentials.

## 1 Introduction

Grape is an economically important and one of the most grown fruits worldwide (1). Most of the production (about 80% of the yield) is used for wine making (2). An important by-product of grape processing – grape pomace is the most important residual after juice extraction or wine making and consists of peel, seed, stem, and pulp (3). Grape pomace is considered a rich source for the extraction of a wide range of valuable phytonutrients, which exhibit a variety of bioactivities, such as antioxidant, anti-inflammatory, cardioprotective, anti-aging, antimicrobial and anti-cancer properties (4–9). Bioactive substances including proanthocyanidins, anthocyanins, phenolic acids, stilbenes, and flavonols are abundant in grape by-products (10) that can help in prevention or management of several conditions such as inflammatory conditions characterized by bowel disruption and the involvement of the immune system and colorectal cancer. Grape by-products can promote remarkable effects in reducing pro-inflammatory, pro-oxidative, and proliferative actions in inflammatory bowel diseases and colorectal cancer both *in vivo* and *in vitro* (10). Moreover, bioactive substances, such as resveratrol (11), anthocyanidins like delphinidin (12) and procyanidin such as procyanidin B2 (13) are valuable in multiple industries, including pharmaceuticals, agri-food, or cosmetics (13–15). Another abundant by-product of winemaking is grapeseed oil, which is processed from grapeseeds and presents an excellent source of γ-tocotrienol, and α-tocopherol. It also contains fatty acids mainly linoleic, oleic, palmitic, and stearic acids, as well as polyunsaturated fatty acids (PUFAs), monounsaturated fatty acids (MUFAs) and saturated fatty acids (SFAs) (16). Furthermore. secondary plant metabolites such as polyphenols are produced by the grape berries during the growth in reaction to environmental stressors. They form significant components of red wines that enhance the sensory qualities and antioxidant capacity (17). Red wine polyphenols comprise newly generated ones during the winemaking process (such as highly polymerized polyphenols) in addition to those found in grapes as mentioned earlier many of which are recognized to possess beneficial impacts on health (17). Although several studies have summarized the most known physiological and therapeutic effects of grapes and their by-products (12, 15, 18–20), the action of grape extract and grape polyphenols on reproductive processes has not been sufficiently elucidated yet. The present evidence-based study summarizes the current knowledge concerning the provenance, properties, as well as physiological and therapeutic actions of grape extract and grape polyphenols on various cellular processes with a focus on female reproduction.

## 2 Provenance, bioactive substances and physiological actions

Grapes (*Vitis vinifera* L.) present an important source of phenolic compounds including phenolic acids, tannins, coumarins, flavonoids, flavones, and stilbenes (7). Grape pomace also contains neutral polysaccharides (30%), pectic substances (20%), and insoluble proanthocyanidins (15%) (21).

Among grape pomace compounds with high nutraceutical value, polyphenols (phenolic acids, flavanols, flavonols, anthocyanins, proanthocyanidins, and stilbenes) are the most interesting due to their bioactive properties (22–24). One of the most efficient bioactive compounds found in grape skin, seeds, and wine is stilbenoid resveratrol (25, 26), widely known primarily for its phytoestrogenic and antioxidant activities (27). Additionally, polyphenolic pigments anthocyanidins, including delphinidin, mainly extracted from grape skins, are responsible for many of the red-orange to blue-violet colors (28). Grapeseeds contain proanthocyanidins, which are composed of epicatechin and monomeric catechin, gallic acid, and polymeric and oligomeric proanthocyanidins (29). Interestingly, proanthocyanidins present more powerful free radical scavengers than vitamins C, E, or β-carotene (30). Monomeric and dimeric flavanols, as well as mono- and diglycosides have been identified in grapeseed extracts. Diglycosylated flavanol dimers have been detected in grape skin extracts, too. The concentration of the mono- and diglycosides depends largely on the grape variety and grape source (31). Major bioactive compounds present in different parts of grape and grape products are given in **Table 1**.

**Table 1:** Major bioactive compounds in different parts of grape and grape products.

Grape part/product	Bioactive compounds	References
Seeds	catechin epicatechin epicatechin-3-O-gallate proanthocyanidins procyanidin B2 dimeric procyanidin gallic acid	[15, 29, 32–36]
Skin	epigallocatechin kaempferol myricetin trans-resveratrol quercetin proanthocyanidins ellagic acid	[15, 32, 33, 37–40]
Leaves	kaempferol myricetin quercetin gallic acid ellagic acid	[32, 41]
Stems	quercetin 3-O-glucuronide rutin astilbin trans-resveratrol	[15, 38, 42, 43]
Raisin	hydroxymethylfurfural hydroxycinnamic acid	[41, 44, 45]
Red wine	catechin cyanidin-3-glucoside peonidin-3-glucoside cetunidin-3-glucoside delphinidin-3-glucoside resveratrol quercetin hydroxycinnamic acid	[43, 45–47]

Grape polyphenols are effective inhibitors of enzymes linked with various ailments. Findings indicate an inverse relationship between the consumption of grapes or grape products and the development of age-related complications including cardiovascular disorders with an estimated 6–7% reduction in deaths from cardiovascular disorders (7). Studies demonstrated biological activities including antioxidant, cardioprotective, anti-cancer, anti-inflammatory, anti-aging, and antimicrobial properties exerted by grape polyphenols such as anthocyanins, flavanols, flavonols, and resveratrol. Chromatographic analysis confirmed the presence of 19 phytochemicals. The prominent compound was catechin followed by gallic acid, caftaric acid, and epicatechin (4–6, 48). Moreover, skin protection, antidiabetic, immunomodulatory and anti-neurodegenerative activities as well as hepatoprotective and neuroprotective effects using phenolic compounds gathered form grape ethanol extract have been reported (11, 15, 18, 48).

Oxidative stress has been associated with the pathogenesis of several chronic diseases and inflammatory processes. Polyphenols are strong antioxidants that act as a defense barrier against free radicals, as well as non-radical oxidants (49). Phenolic acids, stilbenoids, tannins, quinones, coumarins and flavonoids from grapes have the potential to enhance the oxidant capacity of cells stimulating enzymatic expression and reducing the reactive oxygen species (ROS) by either inhibiting their production or by directly scavenging them or via xenobiotic detoxification. For example, administration of Bordo grape juice to human test subjects, led to elevation of antioxidant activities and lowering of blood glucose (50). Grape polyphenols, particularly flavanols can maintain cellular protein homeostasis (proteostasis). Since impaired proteostasis is closely involved in all amyloid diseases, grapeseed extracts may be a valuable therapeutic agent for the prevention and/or management of neurodegenerative diseases (51). The antimicrobial activity against Gram-positive bacteria and antioxidant properties could be associated with phenolic compounds found in grape stems (52). Resveratrol isolated from grape stems was applied on hepatocellular carcinoma Hep-G2 (hepatoma G2) cells, breast adenocarcinoma MCF-7 cells, colon carcinoma HCT116 cells, and lymphoblastic leukemia cells (1301). After treatment, it was shown that resveratrol possesses anti-proliferative and apoptotic effects (53). Anthocyanidins have been found to possess anti-aging and anti- inflammatory properties (28). Lim and Song (12) described the possible use of delphinidin according to its effect on different types of cancers and various chronic diseases. For the study, they used ovarian adenocarcinoma cells (SKOV3) which were then treated with delphinidin alone or with various inhibitors of cell signaling proteins.

Grape pomace contains a high level of antioxidants with the ability to counteract chronic inflammatory symptoms which was demonstrated on colorectal adenocarcinoma-derived intestinal epithelial cell line Caco-2 after grape pomace ethanolic extract treatment (54). Additionally, grapeseeds contain several flavonoids and non-flavonoids which can exert antioxidant and anti-inflammatory activities. Beneficial effects of grapeseed extract in relation to oxidative stress and metabolic disorders such as insulin resistance have been associated with the modulation of plasma adipokines in mammals (55, 56). Grapeseed supplementation has the potential to scavenge oxygen free radicals in the egg yolk in mammals and chicken, as well as it can reduce oxidative damage in the liver in rats (57, 58). It has been reported that grapeseed proanthocyanidin extract possess anti-inflammatory and antioxidant activities (13, 59, 60), and can reduce cytotoxicity as well as genotoxicity (49), including decreasing oxidative damage induced by aflatoxins (61). Proanthocyanidin B2 found in grapeseed present one of the most valuable components of grapeseed extract and can be used due to its protective action against oxidative stress and development of cardiovascular diseases demonstrated on human umbilical vein endothelial cells (HUVEC) (62, 63). In this regard, grape extracts and their polyphenols exhibit protective effects against different toxins and a variety of mechanisms of their action, disturbing physiological homeostasis through increase in superoxide dismutase (SOD) levels and glutathione peroxidase activities, as well as decrease in malondialdehyde (MDA) levels or activation of the nuclear erythroid 2-related factor 2/ARE pathway demonstrated on PC12 rat cells (26). Possible physiological and therapeutic actions of grape polyphenols depending on their bioactive substances are presented in **Table 2**.

**Table 2:** Physiological and therapeutic actions of grape polyphenols.

Action(s)	Bioactive compounds	References
Anti-inflammatory	catechin epicatechin kaempferol resveratrol procyanidin	[34, 39, 40, 47]
Anti-proliferative Anti-cancer	catechin epicatechin-3-O-gallate resveratrol procyanidin B2 malvidin-3-glucoside	[4, 5, 34–36, 41, 45, 64]
Antioxidant	catechin epicatechin-3-O-gallate rutin myricetin kaempferol quercetin resveratrol gallic acid	[39–41, 43, 45, 64, 65]
Neuroprotective	procyanidin B2 malvidin-3-glucoside peonidin-3-glucoside	[35, 45]
Anti-diabetic	epigallocatechin rutin myricetin quercetin	[37, 40, 43]
Anti-bacterial	catechin epicatechin quercetin resveratrol	[65, 66]
Action on female reproductive processes	myricetin resveratrol procyanidin B2 delphinidin	[8, 12, 13, 59, 60, 67–75]

## 3 Effect on female reproductive processes

**Figure 1:**
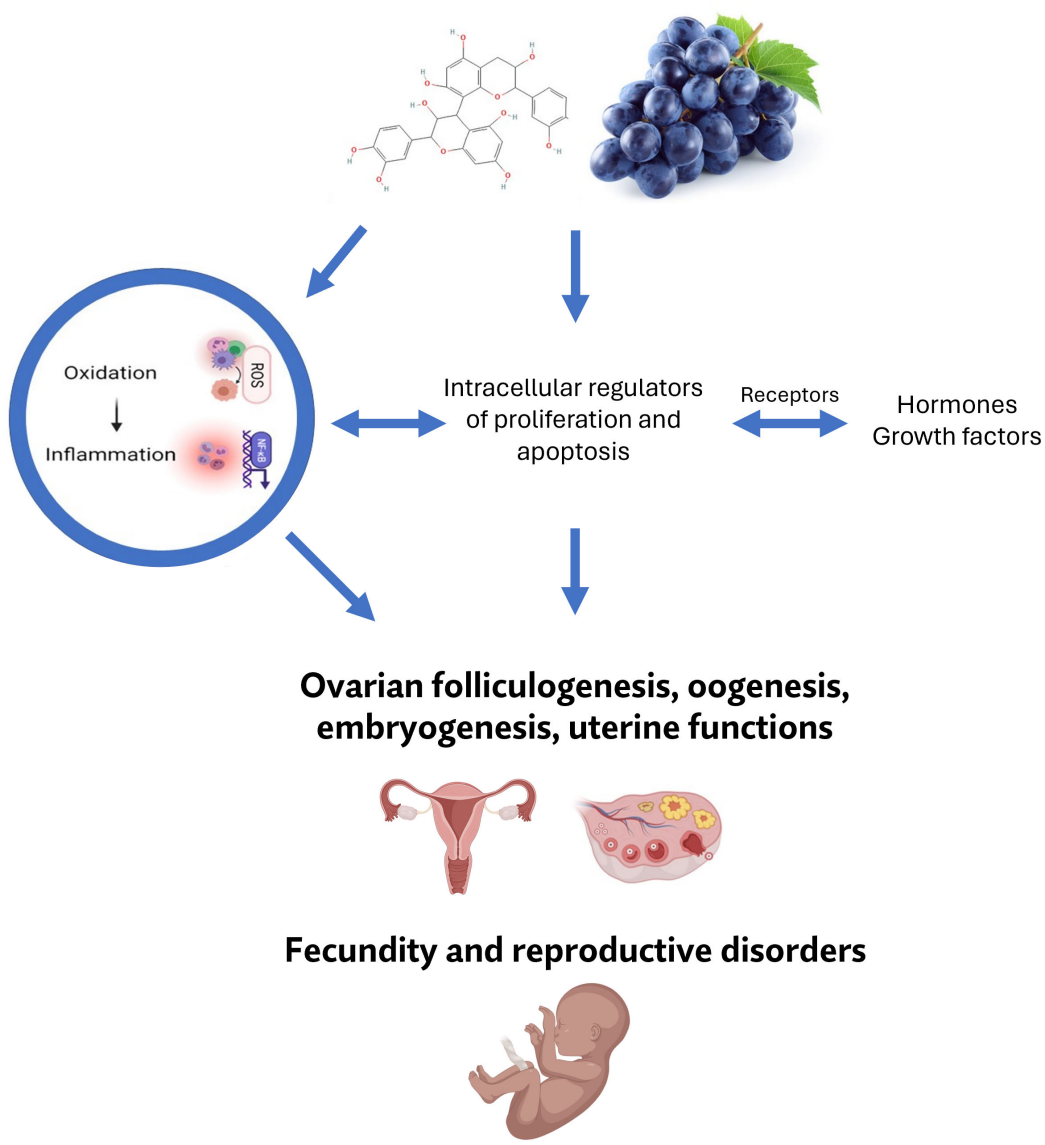
Possible involvement of bioactive constituents of grape (mainly its polyphenols) in female reproductive processes, and their pathological alterations.

Reproductive dysfunctions can be indicated by a negative correlation between muscle growth and reproductive effectiveness (76, 77). Exposure to oxidative stress can lead to the inflammation initiation which is the trigger of multiple reproductive disorders, including ovarian cancer or multiple reproductive defects, such as oocyte mutation, polycystic ovary syndrome (PCOS), endometriosis, as well as can affect ovarian folliculogenesis, oocyte maturation and the release of sex hormones (78–80). Bioactive phytonutrients are known to impart several properties such as anti-inflammatory and antioxidant activities that may have a beneficial impact on reproductive functions (81, 82). Polyphenols can pass through various protective barriers in reproductive organs, which can possibly affect their physiological functions (11, 83, 84). Moreover, grape seed extract had positive impact on improving fertility in golden laying hens (85). Although few studies have been carried out on reproductive cells we have summarized the available information related to the effects of grape polyphenols on female reproductive organs and their (dys)functions in a nutshell. The action on female reproductive processes is presented in **Figure 1**.

### 3.1 Effect on ovaries

Oxidative stress plays an important role in ovarian aging and can lead to decline of fertility in animals and humans (71, 86). According to Shen et al. (87), apoptotic processes induced by oxidative stress in granulosa cells are considered a major cause of follicular atresia. It has been reported that polyphenols can improve the amount and quality of oocytes in mice and humans which was demonstrated after treating oocytes (71, 88). Beneficial effects of grapeseed extract on oocyte maturation and early development based on the mean numbers of cleavage, morula, and blastocyst rates have been observed in sheep (89). Grapeseed procyanidin B2 can positively affect oocyte viability in mice and promote their maturation and developmental capacity (74). The use of grapeseed extract could also be effective in the prevention or treatment of PCOS. Short-term grapeseed extract treatment provided a beneficial impact on PCOS positive women’s metabolic status (90). Furthermore, grapeseed extract can exert a positive impact on health in reproductive insufficiency and menopause and, also prevent negative morphological changes in ovaries due to reproductive ageing (71, 91). This effect could be due to the presence of proanthocyanidin B2, which has been observed in rat ovaries as a possible protection against age-dependent degenerative changes (11, 71). Some studies have described the protective role of proanthocyanidin B2 from grapeseed against damage to rat ovarian tissue induced by ischemia or ischemia/reperfusion (70, 71, 92). Grapeseed extract can affect resistance to chemotherapy and reduce human ovarian cancer cell growth (13). Delphinidin such as a member of the anthocyanidin family and a natural pigment in grapes may be a pivotal therapeutic target for the prevention of epithelial ovarian cancer (12). Grapeseed ethanol extract, as well as proanthocyanidin B2 can modulate human granulosa cell functions, including steroidogenesis, and can exert phytoestrogenic activity with a positive effect on steroid hormone production in human granulosa cells (93). Available data suggest the potential of using maternal diet supplemented with grapeseed extract in the improvement of egg quality in hens. Furthermore, grapeseed extract can ameliorate egg quality by decreasing the rate of double yolk eggs and by improving the size of normal eggs and the elasticity of the shell (60). In contrast, supplementation of dietary polyphenol resveratrol could not impact egg production and egg quality related to the shell, yolk, and albumen in quails (67). In hens, grapeseed proanthocyanidins have been reported to play an important role in the prevention of ovarian aging process by reducing oxidative stress (71).

### 3.2 Effect on uterus

Colitti et al. (94) described the possible impact of grapeseed extract on endometrial functions. In heifers, grapeseed extract (oral administration) affected the expression of several genes in the uterine endometrium. In addition, anti-inflammatory properties of resveratrol present in grapes can contribute to the prevention of endometriosis. This well-known phytonutrient has been considered a novel drug in endometriosis prevention and/or treatment (72, 95). Resveratrol has also been reported to modulate the response of endometrium to progesterone and estrogen during decidualization and reinforce hormone action during human endometrial stromal cell (ESC) differentiation, which could lead to improvement of women’s health (73). Another study provided evidence of promising chemopreventive properties of proanthocyanidins in grapeseeds against cervical cancer. Proanthocyanidin B2 can suppress cervical cancer proliferation and growth and induce apoptosis through the mitochondrial signaling pathway (68). Thus, available literature so far suggests the impact of grape polyphenols on uterine endometrium, decidualization, and their potential to prevent and/or treat endometriosis and cervical cancer. Physiological and therapeutic actions of grape polyphenols on female reproductive processes are presented in **Table 3**.

**Table 3:** Physiological and therapeutic actions of grape polyphenols on female reproductive processes.

Bioactive compound(s)/ extract	Experimental model(s)	Effect(s)	Reference(s)
**Procyanidin B2**	a type 1 diabetes mouse oocytes	reducing oxidative stress; promoting mitochondrial function; improving oocyte quality, maturation, and embryo development	[74]
	porcine granulosa cells	reducing oxidative stress; inhibiting of H_2_O_2_-induced apoptosis	[59]
	ICR mice granulosa cells	improving cell viability; inducing apoptosis; reducing oxidative stress	[70]
**Procyanidin**	human ovarian cancer cells A2780 and A2780/T	enhancing cytotoxicity; suppressing inflammation	[13]
**Proanthocyanidins**	human cervical cancer cells HeLa and SiHa	inhibiting cell growth; inducing apoptosis	[68]
**Grape seed proanthocyanidin extract (GSPE)**	hyline brown hen ovaries	reducing oxidative stress; preventing ovarian aging	[71]
**Lipophilic grape seed proanthocyanidin (LGSP)**	human cervical cancer cells HeLa; HeLa-derived xenograft zebrafish model	increasing ROS production; inhibiting cell growth; inducing apoptosis	[8]
**Myricetin**	bovine granulosa cells (GC) and theca cells (TC)	direct effect on steroidogenesis; reducing of inhibitory effects of mycotoxins	[75]
**Delphinidin**	ovarian cancer cells SKOV3	inhibiting of cell proliferation	[12]
**Resveratrol**	human endometriotic implants in nude mice	inhibiting of endometriosis lesions	[95]
	Sprague-Dawely rat ovaries	protecting and restoring ovarian functions after radiotherapy-induced premature ovarian failure (POF); suppressing of inflammation	[69]
	immortalized human endometrial stromal cell (t-HESC)	enhancing decidualization; decreasing cell proliferation	[73]

## 4 Regulation of female reproductive processes

Grape, grape extract and their bioactive polyphenols can affect female reproductive processes via extracellular regulators and multiple intracellular signaling pathways. Their mechanism(s) of actions on female reproductive processes have been studied insufficiently, however, there are some studies describing the possible mechanism(s) of effect on female reproduction (11).

### 4.1 Hormonal regulation and steroidogenesis

Phenolic compounds present in grapes can affect the essential regulators of reproductive processes, including hypothalamic neurohormones (GnRH, oxytocin, LH and FSH), steroid hormones (estradiol, progesterone, testosterone) and prostaglandins (96). Furthermore, due to the chemical similarity of polyphenols to the structure of estrogens, they may exert hormone-like effects (estrogen-agonistic or antagonistic) by binding or activating estrogen receptors (ERα and ERß) (11, 97). A flavonol myricetin present in red wine can block insulin-like growth factor I (IGF-I)-induced progesterone production by granulosa cells and stimulate IGF-I induced estradiol production (75). Similarly, resveratrol can increase prolactin and IGF-I binding protein 1 (IGFBP1) release, which can result in enhanced decidualization of human embryonic stem cells (ESCs) *in vitro* (73). Grapeseed extract can influence insulin sensitivity by increasing insulin receptors expression and stimulation (98).

Regarding the effect on steroidogenesis, grape extracts, as well as grapeseed proanthocyanidin B2 improved progesterone and estradiol secretion and this was associated with a higher level of the cholesterol carriers, steroidogenic acute regulatory protein (StAR), cyclic adenosine monophosphate response element-binding protein (CREB), and mitogen-activated protein kinases extracellular signal-regulated kinases 1/2 (MAPK ERK1/2) phosphorylation in both primary luteinized human granulosa cells (hGC) and human tumor granulosa cells (KGN). Taken together, GSE and GSPB2 *in vitro* treatments decrease oxidative stress and increase steroidogenesis without affecting cell proliferation and viability in human granulosa cells (93).Another study described the ability of grapeseed extract to modulate an aromatase inhibitor *in vitro* as well as *in vivo* in aromatase-transfected MCF-7 (MCF-7aro) BC xenograft mice (99). Oral administration of grapeseed to heifers can alter progesterone release during estrous cycle after daily oral administration of grapeskin extract for 3 weeks (94). A flavonol myricetin present in red wine can directly affect ovarian function, including steroidogenesis in bovine granulosa cells and theca cells *in vitro*. These cells were gathered from non-pregnant beef cows. Moreover, myricetin has been able to reduce some of the inhibitory effects of mycotoxins on granulosa cell functions (75).

### 4.2 Proliferation and apoptosis

Grape polyphenols may affect ovarian cell functions and physiological processes. Interestingly, it has been demonstrated that grapeseed proanthocyanidin B2 may play an important role in the regulation of apoptosis and proliferation in the ovaries (71). In addition, grapeseed proanthocyanidin B2 treatment can inhibit hydrogen peroxide (H_2_O_2_)-induced apoptosis in granulosa cells possibly via let-7a upregulation, resulting in protective effect and promotion of viability of porcine granulosa cells (59). Furthermore, grapeseed extracts can inhibit Akt phosphorylation, which can regulate multiple cellular processes such as cell proliferation, survival, and metabolism (100). Another grape constituent, delphinidin inhibits ovarian cancer cell proliferation via inactivation of PI3K/AKT and ERK1/2 mitogen-activated protein kinase signaling pathway, which could be a pivotal therapeutic target for the prevention of epithelial ovarian cancer (12).

Lipophilic grapeseed proanthocyanidin can exert an anti-proliferative effect on cervical cancer HeLa cells by increasing ROS production, resulting in the induction of cellular apoptosis, and cell cycle arrest in the G2/M phase. Proanthocyanidin can reduce mitochondrial membrane potential, upregulate Bax/Bcl-2 ratio, increase the release of cytochrome c, and activate caspase-3 and poly(ADP-Ribose)polymerase (PARP), and thus it can induce apoptotic processes in cervical cancer cells through the intrinsic mitochondrial/caspase-mediated pathway (8). Higher concentrations (50 to 100 μg/mL) of grapeseed extract and proanthocyanidin B2 can inhibit cell proliferation in a human ovarian granulosa-like tumor cell line KGN and hGCs, associated with decrease in cyclin D2 level and an increase in p21 and p27 levels to induce cell cycle arrest in G1 phase (93). While proanthocyanidin B2 did not influence nuclear and cytoplasmic apoptosis in porcine granulosa cells (59), it inhibited the ovarian cancer cell viability and enhanced the resistance to chemotherapy (13). Moreover, both grapeseed extract and proanthocyanidin B2 can increase the cleaved caspase-3 level and impair Bcl-2-associated death promoter protein (BAD) phosphorylation, resulting in cell death. Thus, both can inhibit the expression of intracellular markers (MAP kinase, cyclin D2, Akt phosphorylation), and promote the expression of proliferation inhibitors or apoptotic markers (p21, p27) in ovarian granulosa and ovarian cancer cells (13, 93, 101).

### 4.3 Oxidative stress

It is known that oxidative stress is a key promoter of reproductive alterations that can negatively affect ovarian functions through apoptosis induction. Moreover, it can dysregulate the expression of related genes (59, 87). ROS production can lead to oxidative stress affecting ovarian functions. Thanks to its protective properties against oxidative stress, grapeseed proanthocyanidin B2 can prevent ovarian aging by oxidative stress suppression in hens (71). In addition, proanthocyanidins can improve oocyte quality, viability, and maturation, as well as developmental capacity by inhibiting ROS production in murine oocytes (74). Dietary grapeseed extract supplementation can reduce ROS levels in egg yolk suggesting a reduction in both oxidative stress and lipid peroxidation in reproductive broiler hens (60). Additionally, a decrease in lipid peroxidation level and an increase in antioxidant capacity in egg yolk have been observed in laying hens, fed with grape pomace flour (102–104). Furthermore, grapeseed extract and grape polyphenols, such as resveratrol and proanthocyanidin B2 may suppress oxidative stress in non-cancerous and cancerous granulosa cells by promoting antioxidant enzymes (59, 93, 105). Moreover, grapeseed extract may exert a negative prooxidant or beneficial antioxidant effect through modulation of NOX actions (106) and possess the ability to regulate ROS production in human granulosa cells. At low concentrations (0.1 to 10 μg/mL), it can reduce oxidative stress by decreasing ROS content and NOX4 expression (93).

Hypothalamic–pituitary–adrenal axis is activated by stress, which can increase glucocorticoid secretion and disrupt the ovarian cycle (94). Maternal dietary supplementation of grapeseed extract can reduce plasma and tissue oxidative stress associated with the modulation of adipokines content in plasma and peripheral tissues in broiler hens (93).

Regarding the anti-inflammatory activity of grapes, a study indicated that grapeseed extract may reduce the expression of pro-inflammatory interleukins in rats suffering from PCOS (107). Furthermore, resveratrol acts in counteracting the inflammatory signaling pathway associated with radiotherapy-induced premature ovarian failure. Resveratrol has been reported to ameliorate cell damage in ovary induced by ionizing radiation and have a protective effect on endometriosis via downregulation of prostaglandins, interleukins, and stimulating inflammation transcription factor NF-κB (69, 72). Furthermore, resveratrol activates SIRT1 expression, resulting in the inhibition of poly(ADP-Ribose)polymerase-1 (PARP-1) and NF-κB expression-mediated inflammatory cytokines, as well as can restore ovarian function by increasing anti-Müllerian hormone (AMH) levels (69). Similarly, grapeseed procyanidin has demonstrated an inhibitory effect on NF-κB activity and MAPK/ERK pathway mediated YB-1 in ovarian cancer cells, suggesting its potential use as a chemo-sensitizer to overcome multidrug resistance in ovarian cancer patients (13).

Based on available reports, grape extract, and its polyphenols such as resveratrol, proanthocyanidin B2 or delphinidin may be considered to influence female reproductive physiological and pathological processes, as well as regulate multiple signaling pathways related to sex hormones, steroid receptors, intracellular regulators of proliferation, oxidative stress, inflammation, and apoptosis (11).

## 5 Possible application in reproductive biology and medicine

Utilization of grape by-products has attracted increasing attention for the availability of grape skins, their health benefits and pharmacological use. Grape polyphenols can play an important role in the prevention of reproductive disorders due to their ability to mitigate the negative impact of oxidative stress and inflammation on the reproductive processes. Moreover, the beneficial impact on oocyte maturation, cell viability, cell proliferation, as well as steroidogenesis has been reported. Resveratrol from grape stems may have a potential to prevent endometriosis and could serve as a novel dietary supplement. Furthermore, available data suggest the possible use of grapeseed extract to improve oocyte quality, as well as healthy gravidity, embryogenesis, and labour due to its beneficial effect on the endometrium. Moreover, the applicability of grapeseed extract including proanthocyanidin B2 in the prevention and/or management of endometriosis, age-related menopausal reproductive insufficiency and ovarian or cervical cancers has been mentioned. Therefore, grape extract and polyphenols present a promising biostimulator, which can be used as dietary supplement in the improvement of reproduction in the field of animal production, biotechnology, or assisted reproduction. Similarly, phytoestrogenic activity of grape might be used as a potential alternative tool to the hormonal treatment of disorders related to estrogen deficiency, such as menopausal syndrome, and osteoporosis. However, to our knowledge, such potential of grape extract or grape polyphenols has not been examined in depth yet.

## 6 Conclusions and possible directions of future studies

The present review sheds light on the potential health benefits of grape polyphenols while also emphasizing the need for further research and a more cautious interpretation of the findings. It is evident thatconfirmatory claims about the therapeutic effects of grape polyphenols cannot be made at this stage, given the intricacies of human physiology and the many variables at play. Grape polyphenols exert a wide range of health benefits posing grape extract as an interesting and valuable dietary supplement for natural complementary therapy. This evidence-based study focuses on the actions of grapeseed extract and grape polyphenols on female reproductive processes at various regulatory levels and multiple signalling pathways by regulating reproductive hormones (GnRH, gonadotropins, prolactin, steroid hormones, IGFBP), steroid receptors, markers of proliferation and apoptosis. Moreover, the role of grapes in various reproductive disorders, including reproductive insufficiency, PCOS, menopausal syndrome, ovarian cancer or ovarian ischemia has been indicated. Studies also demonstrate the impact of grapeseed extracts or their bioactive constituents (proanthocyanidin B2, resveratrol, delphinidin) on steroidogenesis, oocyte quality and maturation, and developmental capacity. However, lack of knowledge of standardized dosage limits the clinical applications of grapeseed extract despite the wide range of biological and therapeutic potential.

On the other hand, it should be remembered that *in vitro* and *in vivo* studies have been performed with far greater quantities of polyphenols than those frequently found in human diets. Hence, the extent of grape polyphenols consumed on a regular basis is an open question and needs to be addressed in future studies. Determining suitable doses for therapeutic applications remains a critical challenge, as highlighted in the previous sections. The appropriate dosage of grape polyphenols is a key factor in achieving the desired health outcomes, and future research should focus on defining these optimal dosage ranges and accounting for potential variations in individual responses. Moreover, the studies have mainly been performed *in vitro* or *in vivo*, whilst clinical studies are lacking and the efficacy of all grape phytosubstances on reproductive processes has not been tested properly yet.

## Author contributions

Conceptualization: SR, AK; writing – original draft preparation: LK, AK; writing – review and editing: SB, MM, LB, AS, SR; supervision: AK. All authors contributed to the article and approved the submitted version.
